# Unravelling the molecular basis of the dominant negative effect of myosin XI tails on P-bodies

**DOI:** 10.1371/journal.pone.0252327

**Published:** 2021-05-26

**Authors:** Lisa Stephan, Marc Jakoby, Arijit Das, Eva Koebke, Martin Hülskamp

**Affiliations:** 1 Botanical Institute, Biocenter, Cologne University, Cologne, Germany; 2 Faculty of Medicine, Institute of Medical Statistics and Computational Biology & Institute for Diagnostic and Interventional Radiology, University Hospital Cologne, Cologne, Germany; Iwate University, JAPAN

## Abstract

The directional movement and positioning of organelles and macromolecules is essential for regulating and maintaining cellular functions in eukaryotic cells. In plants, these processes are actin-based and driven by class XI myosins, which transport various cargos in a directed manner. As the analysis of myosin function is challenging due to high levels of redundancy, dominant negative acting truncated myosins have frequently been used to study intracellular transport processes. A comparison of the dominant negative effect of the coiled-coil domains and the GTD domains revealed a much stronger inhibition of P-body movement by the GTD domains. In addition, we show that the GTD domain does not inhibit P-body movement when driven by a hybrid myosin in which the GTD domain was replaced by DCP2. These data suggest that the dominant negative effect of myosin tails involves a competition of the GTD domains for cargo binding sites.

## 1 Introduction

The plant actin cytoskeleton provides a cellular infrastructure, which enables controlled distribution of cellular components and serves as a scaffold for cell morphogenesis [1–5]. The actin structure continuously adapts to environmental and developmental cues to direct transport to sites of cellular growth, enabling proper cell development, control of cell polarity, and defense against pathogens [3,4,6,7]. Transport along the actin filaments of plant cells is mediated by myosins in a directed and energy-dependent manner [8,9]. In *Arabidopsis thaliana*, the existing 17 myosins are grouped into two classes [10]: myosin class VIII, whose 4 members are involved in endocytosis, cell wall formation and plasmodesmata-mediated transport; and myosin class XI, whose 13 members (XI-1, XI-2 and XI-A to XI-K) are functioning in organelle trafficking, positioning and cytoplasmic streaming [8,9,11–21].

Class XI myosins have a distinct protein domain organization [22] with an N-terminal motor domain, which mediates ATP hydrolysis, several copies of calmodulin-binding IQ motifs, and a C-terminal tail region. The tail comprises an α-helical coiled-coil region (CC) for dimerization of myosins and the globular tail domain (GTD) for cargo-binding. The binding of myosin GTDs to membranous cargos has been found to be mediated by specific anchor proteins, like the myosin receptors of the MyoB, MadA and MadB families for certain classes of vesicles [23–26], and WPP DOMAIN-INTERACTING (WIT) proteins for the nucleus [27]. In contrast, membrane-less mRNA processing bodies (P-bodies) are connected to myosins by direct interaction of the GTD with the P-body core component DECAPPING PROTEIN 1 (DCP1) [28]. Interestingly, most of these anchors bind various myosins, indicating a low myosin-cargo specificity [29]. Only WIT1 and WIT2 were found to exclusively bind myosin XI-I, indicating a unique function of this isoform in controlling nuclear shape and movement [27].

Most of the class XI myosin single mutants have no obvious morphological loss-of-function phenotypes, indicating a high level of redundancy [17,18,30,31]. Only *xi-k* single mutants show a weak distorted phenotype in vegetative tissues [32], which is thought to be due to its specific role in mediating fast and long-range movement of vesicles, peroxisomes, Golgi, mitochondria, and P-bodies [17,24,25,33], as well as its role in actin organization [34,35]. Apart from myosin XI-K, myosins XI-1, XI-2 and XI-I have been found to be involved in organelle trafficking in vegetative tissues [8,9,16–18].

**Fig 1:**
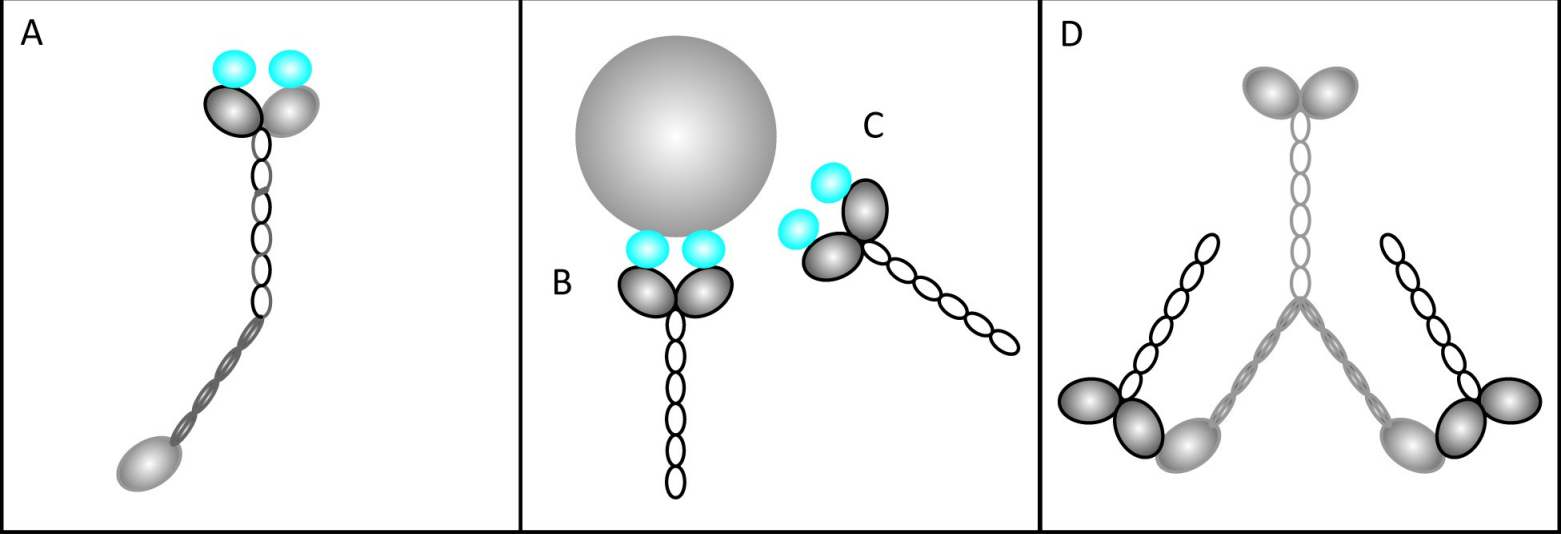
Schematic illustration of hypotheses for the dominant negative effect of truncated class XI myosins on organelle movement. **(A)** Myosin tails dimerize with endogenous myosins and form non-functional, “one-legged” dimers [38]; **(B)-(C)** Myosin tails block adaptor proteins at the cargo **(B)** or in the cytoplasm **(C)** [38]; **(D)** Myosin tails de-activate endogenous myosins by binding to their motor domains [40]. Endogenous myosins are depicted in light grey, truncated tails are shown in dark grey.

In contrast to the minimal effects of null mutations, overexpression of truncated myosin XI tails, lacking the motor domain, has a strong dominant negative effect on cargo movement [9,17,33,36,37]. To date, the molecular reasons for this effect, and consequently essential insights in myosin function, are not known. Several hypotheses have been discussed (Fig 1) [16]: Most commonly, it has been assumed that the truncated myosin tails dimerize with endogenous myosins and form non-functional dimers (Fig 1A) [38]. A second hypothesis is that myosin tails block cargo adaptor proteins on the organelle surface or in the cytosol, thereby preventing organelle binding (Fig 1B and 1C) [38]. Third, it was proposed that the tail fragments bind to the motor domain of endogenous myosins, thereby inactivating them. This hypothesis was postulated under the assumption that a self-regulatory mechanism is operating as described for myosin class V members (Fig 1D) [39–41]. For class V myosins it has been shown that GTDs directly interact with their own motors, thereby causing a conformational change. Due to their interaction, the motor function and the cargo binding are inhibited, rendering the myosin inactive. The three hypotheses are not mutually exclusive and may operate in parallel.

In this study, we show that overexpression of the GTD domain is much more efficient in the inhibition of P-body movement then the coiled-coil domain. We further show that expression of a myosin-DCP2 fusion induces P-body movement and that this movement is not suppressed by expression of the GTD domain, suggesting that the competition for cargo binding sites is important for the dominant negative effect.

## 2 Material and methods

### 2.1 Plant lines and growth conditions

*Arabidopsis thaliana* SALK lines SALK_134363 (*xi-1*), SALK_127984 (*xi-2*), and SALK_92443 (*xi-i*) [42], as well as Gabi-Kat line GK_164_D12 (*xi-k*) [43] were obtained from NASC (Nottingham Arabidopsis Stock Centre) and verified by genotyping. The *Arabidopsis thaliana* triple knockout 3KO (*xi-1* SALK_019031, *xi-2* SAIL_632_D12, *xi-k* SALK_067972) was kindly provided by Valera Peremyslov. Plants were grown on soil or surface-sterilized and grown on ½ MS plates [44]. Seeds were stratified for at least three days and subsequently transferred to long-day conditions at 21±1°C and 100±20 μmol/m^2^s light intensity.

### 2.2 Sequence analysis and plasmids

Sequences were taken from TAIR [45] and NCBI (National Centre for Biotechnology Information, www.ncbi.nlm.nih.gov). Conserved domains were determined using CD-Search [46] and PROSITE [47]. *In silico* sequence analysis was carried out with CLC DNA Workbench version 5.6.1. All constructs used in this study were confirmed by sequencing (GATC/Eurofins, Ebersberg).

Coding sequences of *XI-1* (AT1G17580), *XI-2* (AT5G43900), *XI-I* (AT4G33200), *XI-K* (AT5G20490), *DCP1* (AT1G08370), *DCP2* (AT5G13570), *DCP5* (AT1G26110), *VCS* (AT3G13300), and *XRN4* (AT1G54490), were amplified from Col-0 cDNA. A list of primers is displayed in S1 Table. Primers for CFP, including the coding sequence of LifeAct in the forward primer, were used to amplify LifeAct-CFP and introduce it into the plant expression vector pBATL-eGFP (kindly provided by Phillip Thomas) by restriction/ligation, creating pBATL-LifeAct-CFP. The expression vector pMDC32:XI-K:YFP (XI-K:YFP) was published previously [48]. To create the myosin hybrid constructs (XI-K_ΔGTD_-GOI), the GTD domain of XI-K:YFP was replaced by a Gateway cassette, and the ORF of the gene of interest was introduced by LR cloning. Gateway vectors pENSG:YFP, pEXSG:CFP [49] and pAMARENA (NCBI:txid905036) were used for expression/localization. Expression of plasmids was carried out in rosette leaves of two week old Arabidopsis seedlings by biolistic transformation [50] and analyzed by confocal laser scanning microscopy after 12 to 16 h.

### 2.3 Microscopy

Confocal laser scanning microscopy was carried out with the Leica DMRE, DM5500, and DM6000 Microscopes, and documented with the TCS-SP2 (HCxAPO L40x0.8 water-immersion objective), TCS-SPE (ACS APO 20.0x0.60 water-immersion objective) and TCS-SP8 (HC PL APO CS2 20x0.75 water-immersion objective) imaging systems, respectively (Leica Microsystems, Heidelberg, Germany). CFP was excited at 405 nm, and emission was detected between 460 and 480 nm. YFP was excited at 514 nm, and emission was detected between 530 and 570 nm. mCherry was excited at 561 nm, and emission was detected between 600 and 635 nm. Sequential scanning between frames was used to avoid cross talk between different fluorescently tagged proteins.

### 2.4 Analysis of fluorescence intensity

Confocal pictures of transiently transformed Col-0 rosette leaf epidermal cells were taken at fixed laser intensity and gain, to guarantee comparability of measurements within sets. Whole cells were scanned at fixed Z steps of 1.5 μm at a resolution of 1024x1024 pixels. Z stacks displaying the average intensity were created and analyzed using ImageJ (Fabrice Cordelieres, Institut Curie, Orsay, France). The integrated density was used as a measurement of fluorescence intensity of the cells, which was normalized against the background intensity. Cell areas were determined manually using the polygon tool.

### 2.5 Analysis of P-body movement

The movement of P-bodies was analyzed in transiently transformed Arabidopsis rosette leaf cells along the central vein. A particular plane was scanned 50 times at intervals of 1.29–1.6 sec at a resolution of 512x512 pixels with a confocal laser scanning microscope. Organelle tracking was carried out using the Manual Tracking Plugin for ImageJ. Statistical analysis was carried out with R Studio. In order to assure a robust statistical analysis, putative measurement errors of the manual data were de-noised using an FFT filter. Significance was determined by a pairwise two-sample Wilcoxon test with multiple testing correction at *p*<0.001, *p*<0.01, and *p*<0.05. To analyze the data with regard to the different types of movement, three pairwise statistically different classes of P-body movement were determined. For this purpose, Col-0 P-bodies were divided into three groups by k-means clustering, based on the speed of a trajectory, which is defined as the average speed in each individual step. Subsequently, we identified immobile/slow [0, 0.885), medium [0.885, 1.93), and fast [1.93, 4.39] moving groups using a one-sided t-test.

## 3 Results

### 3.1 Myosin XI GTD and CC localization

**Fig 2:**
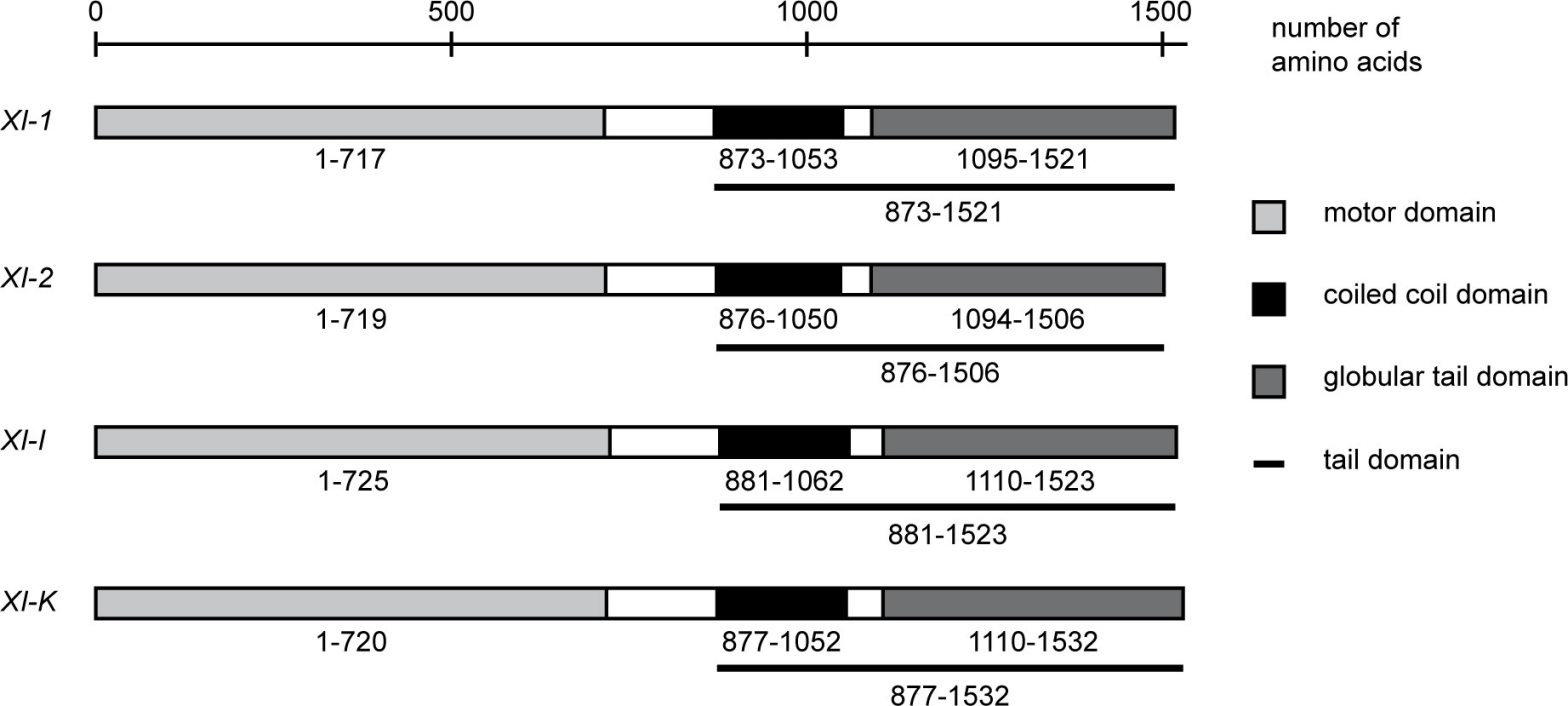
Domain structure of class XI myosins. All myosin fragments used in this work were designed according to the domain structure. Sequences were taken from TAIR [45] and NCBI (National Centre for Biotechnology Information, www.ncbi.nlm.nih.gov). Conserved domains were determined using CD-Search [46] and/or PROSITE [47].

Various studies have shown that myosin XI tails mainly localize to organelles and rarely to the actin cytoskeleton [37,41,51]. Hence, it is likely that the dominant negative effect of these tails is mostly mediated at the sites of their cargos, rather than the actin cytoskeleton. In order to map the functional domain that mediates the dominant negative effect, we fused YFP to the N-terminus of the coiled-coil and GTD fragments of myosins XI-1, XI-2, XI-I, and XI-K (Fig 2) and transiently expressed these fusion proteins under the control of the 35S promoter in Col-0 leaf epidermis cells.

**Fig 3:**
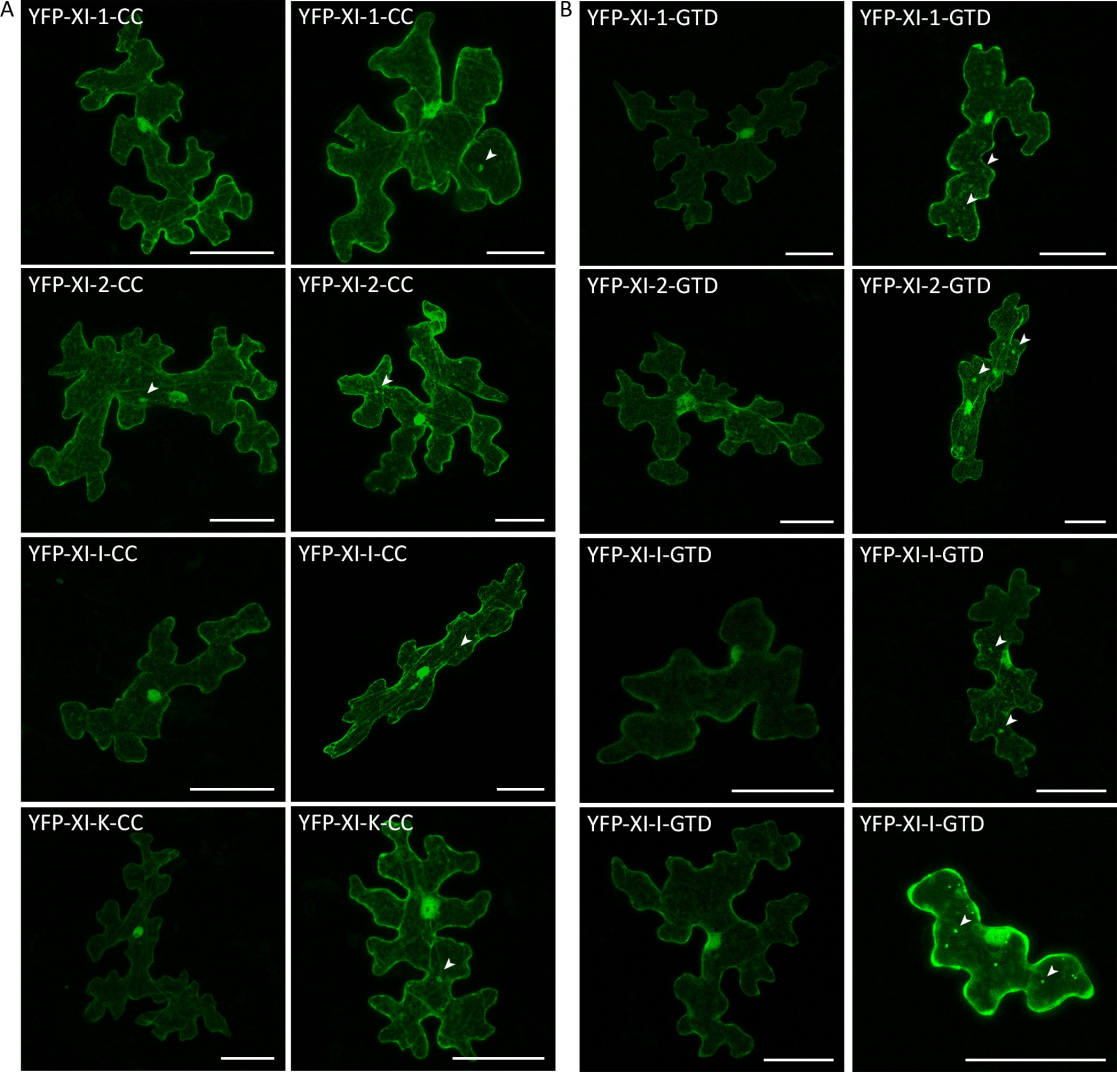
Localization of myosin fragments in Arabidopsis epidermal cells. Transient expression of **(A)** myosin coiled-coils and **(B)** myosin GTDs in Col-0 epidermal pavement cells. The first columns display representative cells showing mainly cytoplasmic localization, the second columns display representative cells showing dot-like structures and small aggregates, indicated by arrow heads. The scales display 50 μm.

First, we analyzed the localization of the eight fragments individually (Fig 3). We found that all transiently expressed coiled-coils and GTDs localize to the cytoplasm, the nucleus and occasionally to some filamentous structures which might represent the actin cytoskeleton or transvacuolar strands (Fig 3A and 3B, first columns). For the coiled-coil fragments, we rarely found dot-like structures or small aggregates (Fig 3A, arrow heads). The GTD fragments showed a more frequent localization in dots (Fig 3B, arrow heads). We analyzed the correlation of fluorescence intensity and occurrence of dots exemplarily for the XI-K-GTD and found that the number of dots increased with the expression strength (S1 Fig).

**Fig 4:**
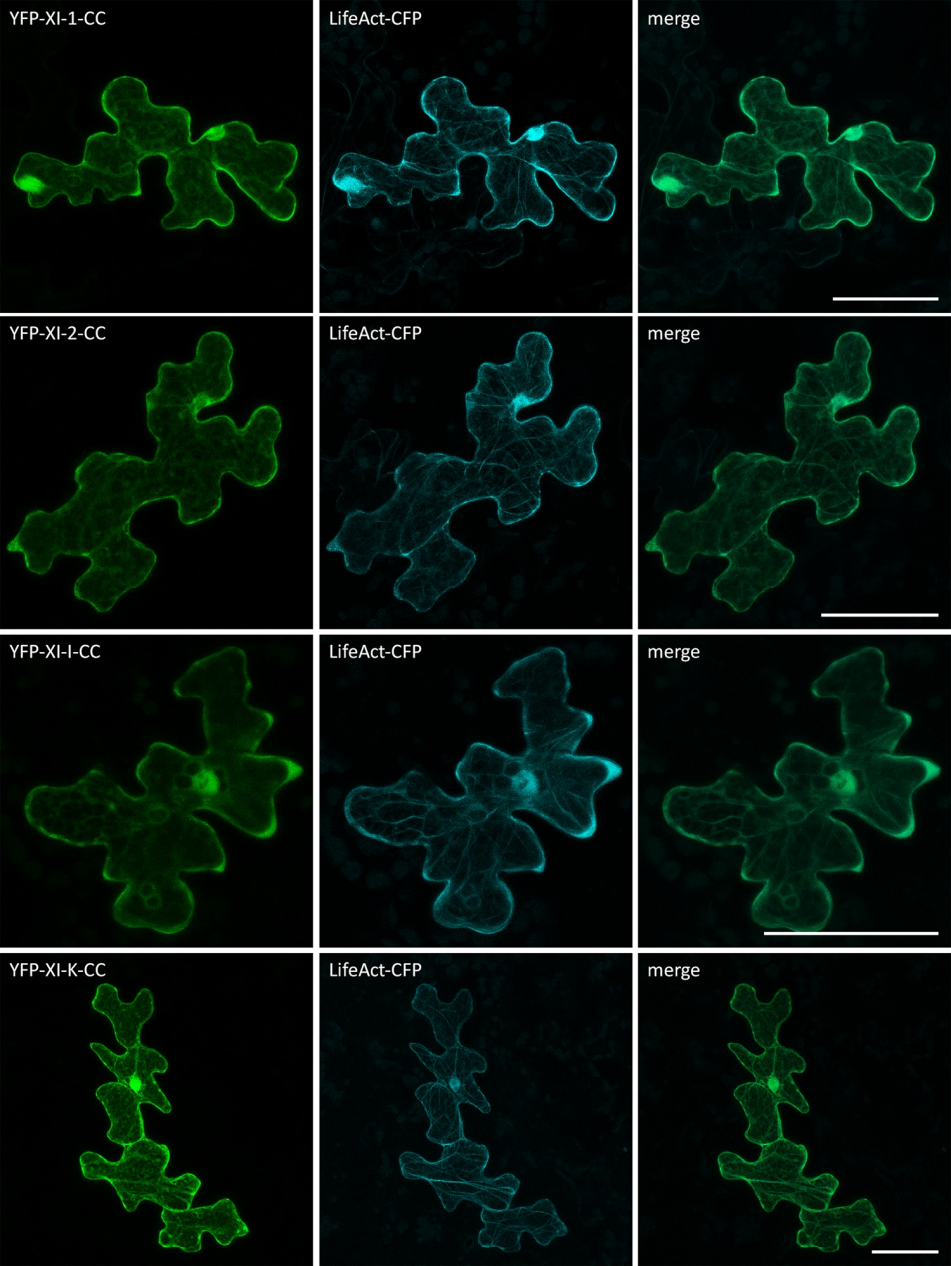
Co-localization of myosin coiled-coils and LifeAct in Arabidopsis epidermal cells. Transient expression of LifeAct-CFP with myosin coiled-coils in Col-0 epidermal pavement cells. The scales display 50 μm. The GTDs did not show any co-localization with LifeAct-CFP (Fig 5).

Subsequent co-localization experiments of the myosin fragments and the actin marker LifeAct-CFP confirmed that some of the filamentous structures labelled by the myosin coiled-coils are indeed actin filaments (Fig 4).

**Fig 5:**
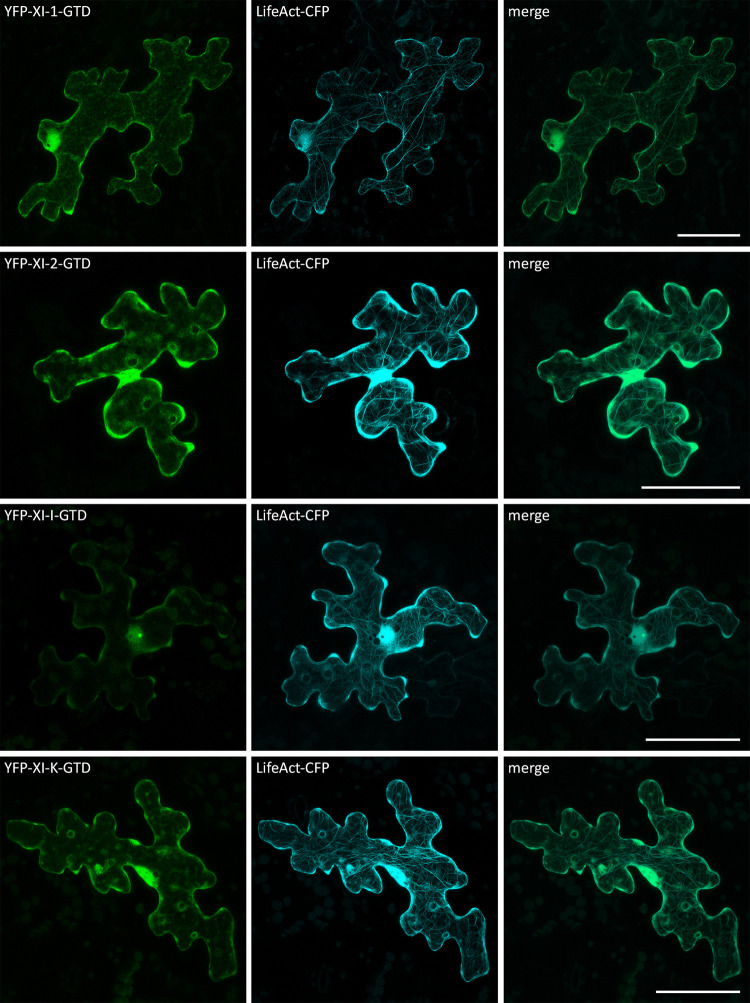
Co-localization of myosin GTDs and LifeAct in Arabidopsis epidermal cells. Transient expression of LifeAct-CFP with myosin GTDs in Col-0 epidermal pavement cells. The scales display 50 μm.

**Fig 6:**
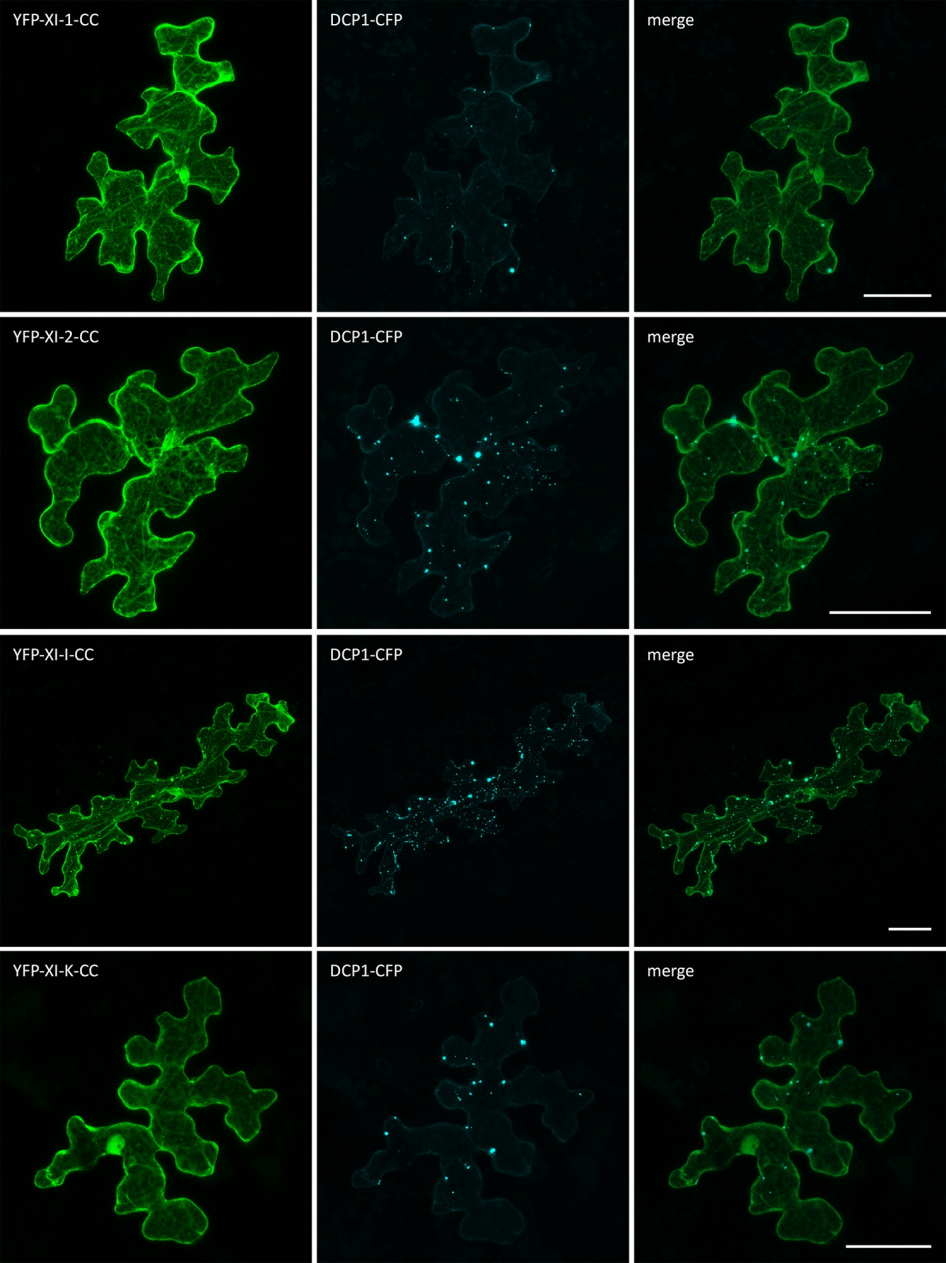
Co-localization of myosin coiled-coils and DCP1 in Arabidopsis epidermal cells. Transient expression of DCP1-CFP with myosin coiled-coils in Col-0 epidermal pavement cells. The scales display 50 μm.

**Fig 7:**
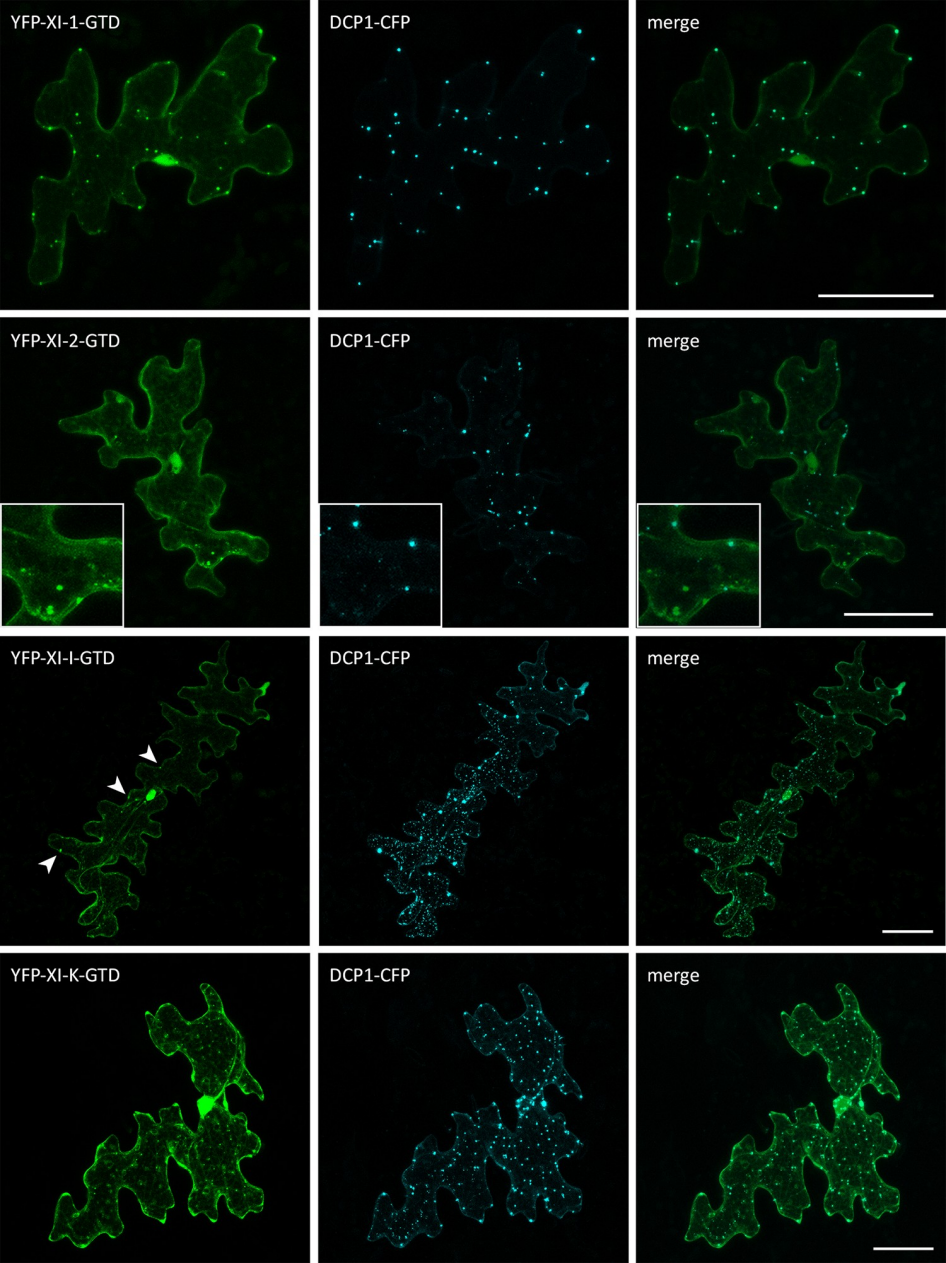
Co-localization of myosin GTDs and DCP1 in Arabidopsis epidermal cells. Transient expression of DCP1-CFP with myosin GTDs in Col-0 epidermal pavement cells. Arrow heads indicate dot-like structures. The scales display 50 μm.

Finally, we performed a co-localization analysis of the myosin fragments and the P-body marker DCP1-CFP (Figs 6 and 7). For all coiled-coil fragments, the occasionally occurring dot-like structures frequently co-localized with DCP1. This was unexpected and may be explained by the formation of non-functional dimers of the coiled-coil domain and endogenous myosins at P-bodies (Fig 6).

The localization of GTDs to P-bodies was very clear for myosins XI-1, XI-I and XI-K (Fig 7, arrow heads). XI-2-GTD expressing cells exhibited only few dots that typically did not co-localize with DCP1(Fig 7, zoom).

### 3.2 The dominant negative effect is mainly mediated by the globular tail domain

**Fig 8:**
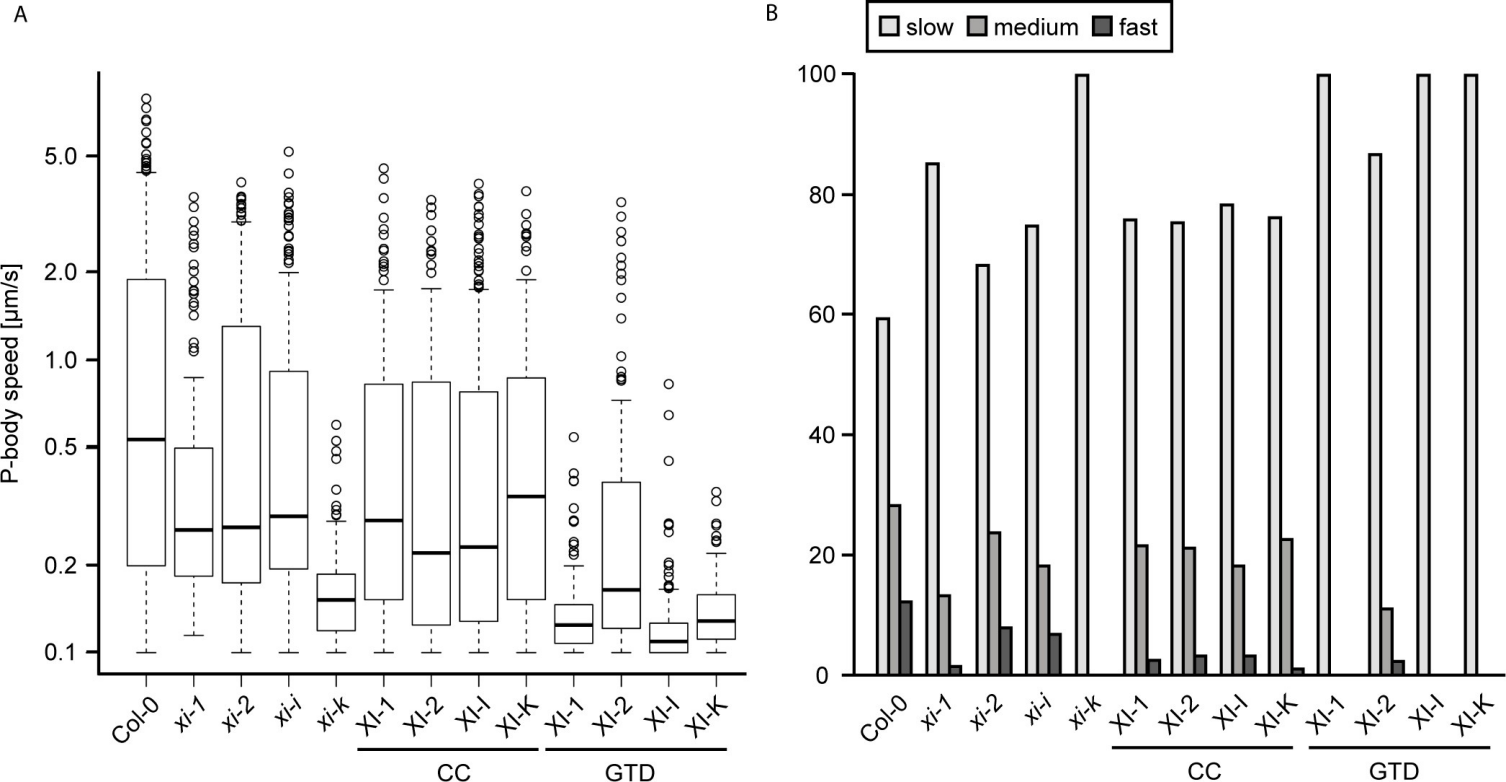
Motility of P-bodies in myosin single mutants and under the dominant negative effect of myosin fragments in *Arabidopsis thaliana* leaf midvein cells. **(A)** Speed boxplot and **(B)** Speed classes of manually tracked DCP1-marked P-bodies in myosin single mutants or in the presence of myosin fragments in Col-0 in percent. Significance was determined by a two-sample Wilcoxon test at *p*<0.001, *p*<0.01, and *p*<0.05 (S2 Table).

The dominant negative effect of myosin fragments on P-body motility was quantitatively compared. Towards this end, we tracked DCP1-CFP marked P-bodies in Col-0 leaf vein cells and compared the speed with cells co-expressing the respective coiled-coil and GTD constructs. As a reference for our analysis, we included the four corresponding myosin *xi* mutants in the analysis (Fig 8, S2 Table). As reported before [28], also for other organelles [30,33], the P-body speed was slightly reduced in the myosin mutants *xi-1*, *xi-2*, and *xi-i* and strongly reduced in *xi-k*. A more detailed analysis of slow, medium and fast moving classes of P-bodies revealed that in *xi-k* mutants the class of fast and medium speed P-bodies is absent.

Co-expression of coiled-coil domains of all four myosins affected P-body speed only weakly, though statistically significant. The effect of XI-1-CC and XI-2-CC resulted in similar P-body speeds as in the *xi-1* and *xi-2* single mutants, respectively. For XI-I, the effect of the coiled-coil domains was slightly stronger than in the respective mutant. The inhibition of P-body speed by XI-K-CC was comparable to the other three coiled-coil constructs and significantly weaker than the effects of a *XI-K* knockout.

In contrast, the GTD domains caused a substantial reduction of P-body speed (Fig 8A) and a total loss of fast and medium speed P-bodies by the GTDs of XI-1, XI-I, and XI-K (Fig 8B). The effect of XI-2-GTD was less pronounced and the medium and fast speed classes were still present. This is consistent with our observation that the XI-2-GTD domain shows almost no co-localization with P-bodies. These results suggest that the dominant negative effect is mainly mediated by the globular tail domains.

### 3.3 Mobilization of P-bodies by myosin-DCP2 fusions is not suppressed by overexpressed GTDs

**Fig 9:**
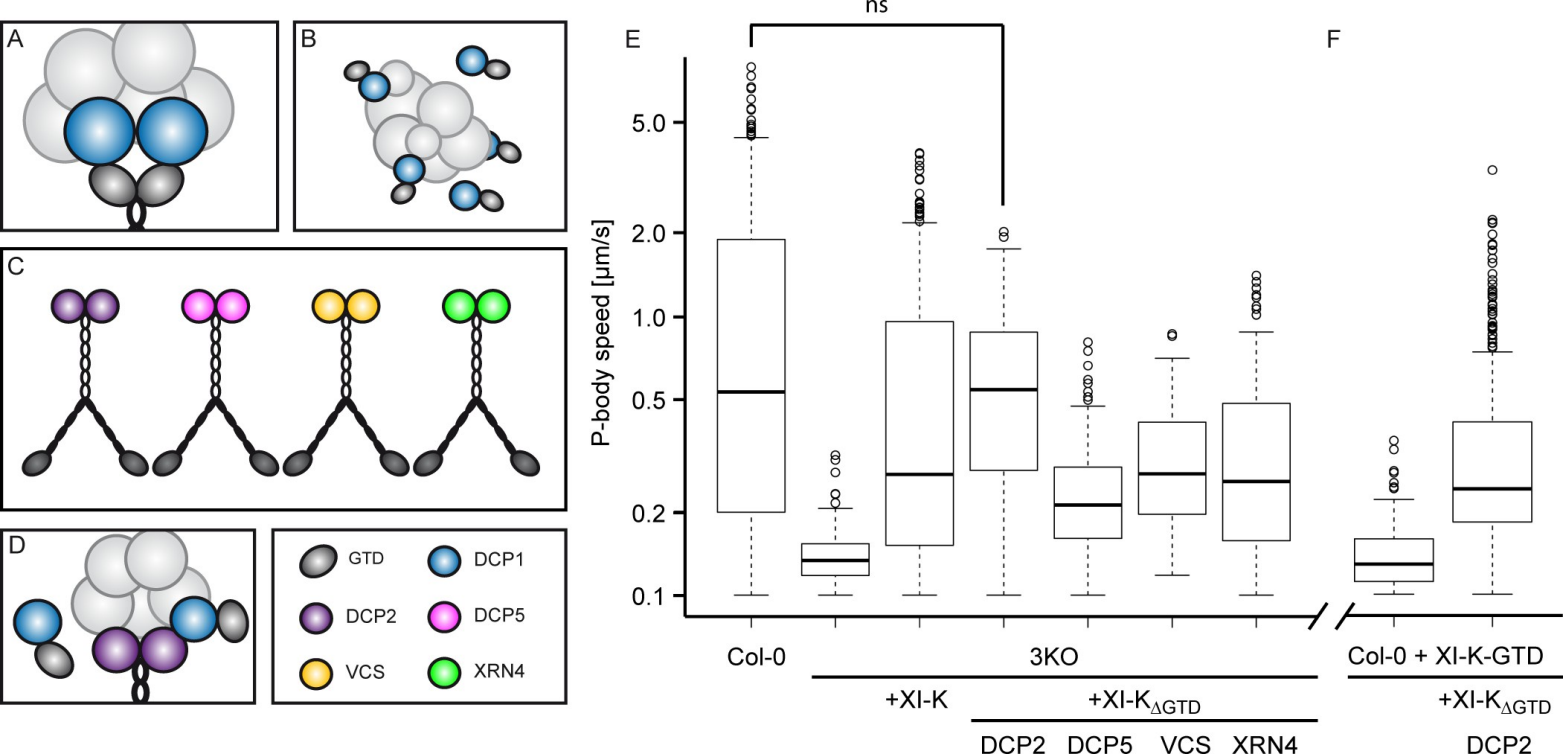
Direct target binding of myosins to test the dominant negative GTD function. **(A)** P-body binding to class XI myosins is mediated by DCP1 [28]. **(B)** DCP1 is blocked by free GTDs on P-bodies or in the cytoplasm. **(C)** The GTD of a full-length genomic myosin XI-K construct under the endogenous promoter [48] was exchanged with the ORF of P-body core components. **(D)** XI-K_ΔGTD_-DCP2 can bind and move P-bodies in presence of free GTDs. **(E), (F)** Boxplot of P-body speed data collected by manual tracking of DCP1-CFP marked P-bodies in transiently transformed cells. Significance was determined by a two-sample Wilcoxon test at *p*<0.001, *p*<0.01, and *p*<0.05 (S3 Table).

To explore whether the expression of GTDs inhibits mobility of P-bodies by competing with full-length myosins for binding to P-bodies, we created a set of myosin hybrid constructs, in which the GTD domains of XI-K were replaced by P-body components (Fig 9). This should connect the myosin directly to the P-body, thereby enabling its mobilization by the fusion protein. In this situation, overexpressed GTDs cannot compete and should not be able to inhibit P-body motility.

XI myosins bind to P-bodies through interaction of the GTD domain and DCP1 (Fig 9A) [28]. Expression of GTD domains leads to a competition of binding to DCP1 with the endogenous XI myosins (Fig 9B). We used a construct expressing the full-length genomic myosin XI-K under the endogenous promoter (XI-K:YFP) [48] that was shown to be capable to rescue organelle motility in the 3KO mutant [34]. In this construct, we replaced the GTD domain with the ORFs (including a C-terminal stop codon) of the P-body core components DECAPPING PROTEIN 2 (DCP2), DECAPPING PROTEIN 5 (DCP5), VARICOSE (VCS), and EXORIBONUCLEASE 4 (XRN4): XI-K_ΔGTD_-DCP2, XI-K_ΔGTD_-DCP5, XI-K_ΔGTD_-VCS, and XI-K_ΔGTD_-XRN4 (Fig 9C). The assembly of P-bodies around these hybrid myosins cannot be disturbed by free GTDs, however, GTDs could still inactivate these hybrid myosins by binding to the motor domains (Fig 9D).

The functionality of the constructs was verified by transient transformation of epidermal pavement cells of the 3KO mutant [34] and subsequent analysis of P-body motility by manual tracking of DCP1-CFP. P-body movement in Col-0, 3KO, and 3KO rescued with XI-K:YFP were included as controls (Fig 9, S3 Table).

The XI-K_ΔGTD_-DCP2 construct showed a full rescue of the 3KO motility phenotype, which even exceeded the effect of full-length XI-K. The partial rescue by XI-K_ΔGTD_-DCP5, XI-K_ΔGTD_-VCS and XI-K_ΔGTD_-XRN4 was comparable to XI-K:YFP. We therefore decided to use XI-K_ΔGTD_-DCP2 for further analyses.

Next, we investigated the ability of XI-K_ΔGTD_-DCP2 to rescue the dominant negative effect of GTD domain overexpression in the wild type. We co-expressed the two constructs together with DCP1-CFP as a P-body marker. The additional expression of XI-K_ΔGTD_-DCP2 had no significant effect on the expression strength of the GTD and DCP1, which in turn showed a clear positive correlation (S2 Fig). As shown in Fig 9F, P-body motility was clearly restored in GTD expressing cells upon co-expression of XI-K_ΔGTD_-DCP2. These results support the idea that GTD domains exert their dominant negative effect by competition with the full-length myosins for target binding.

## 4 Discussion

Myosin tails have been used as genetic tools to investigate myosin-cargo relations in various studies, but were found to exert their dominant negative effect on organelle motility non-selectively on all organelles [13,33,37]. In the following we discuss three scenarios explaining the molecular mechanism of the dominant effect.

The first scenario suggests the dominant negative effect by the formation of non-functional dimers. In this context it has been a point of debate how non-functional dimers of any class XI myosin isoforms other than XI-K can cause a strong direct dominant negative effect, since the corresponding single mutants do not show a similar phenotype [16]. One possible explanation is that the non-functional dimers block the function of endogenous XI-K at the actin cytoskeleton. However, the tail [37,41,51] and coiled-coil fragments show little co-localization with actin. Another explanation could be that myosin tails of other isoforms form heterodimers with XI-K, similar to other systems [9,52]. However, heterodimers of myosins were only detected in low percentage in some systems and it was shown that homodimers are more thermodynamically stable [52], making it very unlikely that heterodimerization of non-functional dimers could cause a strong dominant negative effect. Consistent with this, we show in this study that overexpression of coiled-coil domains has only a weak effect on P-body movement. In contrast to myosins 1, 2 and I, the dominant negative effect caused by the coiled-coil domain of XI-K was significantly weaker than the loss-of-function phenotype in *xi-k* mutants, suggesting that non-functional dimers are not the main reason for the dominant negative effect. However, one has to consider in this context that cargo binding stabilizes the dimerization of myosins and that the strength of the dominant negative effect mediated by coiled-coil overexpression could be underestimated for this reason [53].

Our finding that the overexpression of all four globular tail domains causes strong repression of movement is consistent with hypothesis two and three: a competition for cargos and an inhibition by binding and blocking the motors. The latter is supported by the finding that the replacement of two amino acids, hypothesized to mediate the interaction between GTD and the motor domain (Fig 1D) [41], was found to abolish the dominant negative effect of the tail domain of XI-K [40]. While this suggests the dominant negative effect is due to an in-activation of the myosin motor, these mutations may also effect cargo binding, targeting, or overall folding of the GTD domain. In this context, it is noteworthy that myosin Va cargo binding was assumed to shift the conformational equilibrium toward the active state. However, it was deemed unlikely that cargo molecules always disrupt the inhibited form [39]. Given the dynamic nature of P-body assembly and disassembly, it is also not likely that the hybrid myosin is constantly bound. Although we cannot exclude that our fusion constructs possess conformational changes which might hamper the interaction of the GTD with the motor domains, the finding that myosin-DCP2 fusion proteins can rescue the dominant negative effect of overexpressed GTD domains renders the latter scenario unlikely. We therefore favor that the inhibitory effect of overexpressed GTD domains is mainly caused by their competition with endogenous myosins for cargo binding sites. Since GTD domain accumulation at P-bodies occurs only in a fraction of cells, but effectively inhibits motility in all cells, we hypothesize competition to occur at adaptors at P-bodies as well as free adaptors in the cytosol.

Our finding that the GTD domains of XI-1, XI-I, and XI-K display a much stronger effect on P-body movement and a stronger co-localization than XI-2, offers the possibility to apply similar approaches for other organelles and/or myosins to screen for specific cargos of a myosin of interest or vice versa, as was initially intended for myosin tail constructs [37]. Finally, the creation of more myosin-anchor hybrid constructs, which exclusively target one organelle, might help to understand the contribution of directed transport to cell function and growth.

## Supporting information

S1 Fig Expression and localization of XI-K-GTD. **(A)** Expression intensity and number of dot-like structures of the GTD construct was measured in Col-0 midvein cells transiently expressing YFP-XI-K-GTD.
(PDF)

S2 Fig Expression and localization of XI-K-GTD. Expression intensity of XI-K-GTD and DCP1 was measured in Col-0 midvein cells transiently expressing YFP-XI-K-GTD and DCP1-CFP with or without XI-K_ΔGTD_-DCP2. The error bars depict the standard deviation. Significance was determined with a two-sided t-test at *p*<0.001 (***), *p*<0.01 (**), and *p*<0.05 (*).
(PDF)

S1 Table Primer list. (PDF)

S2 Table Wilcoxon test with multiple testing correction of P-body motility in myosin single mutants and under the dominant negative effect of myosin fragments in *Arabidopsis thaliana* leaf midvein cells. Significance was determined with a two-sample test at *p*<0.001 (***), *p*<0.01 (**), and *p*<0.05 (*).
(PDF)

S3 Table Wilcoxon test with multiple testing correction of P-body speed data, collected by manual tracking of DCP1-CFP marked P-bodies in transiently transformed *Arabidopsis thaliana* leaf midvein cells. Significance was determined with a two-sample test at *p*<0.001 (***), *p*<0.01 (**), and *p*<0.05 (*).
(PDF)
