# Supplementary material for: Unravelling the molecular basis of the dominant negative effect of myosin XI tails on P-bodies
Source: PLoS One. 2021 May 26;16(5):e0252327. doi: 10.1371/journal.pone.0252327 (PMC8153422; doi:10.1371/journal.pone.0252327)
Supplement: S1 Fig — (A) Expression intensity and number of dot-like structures of the GTD construct was measured in Col-0 midvein cells transiently expressing YFP-XI-K-GTD. (PDF) [file pone.0252327.s001.pdf]

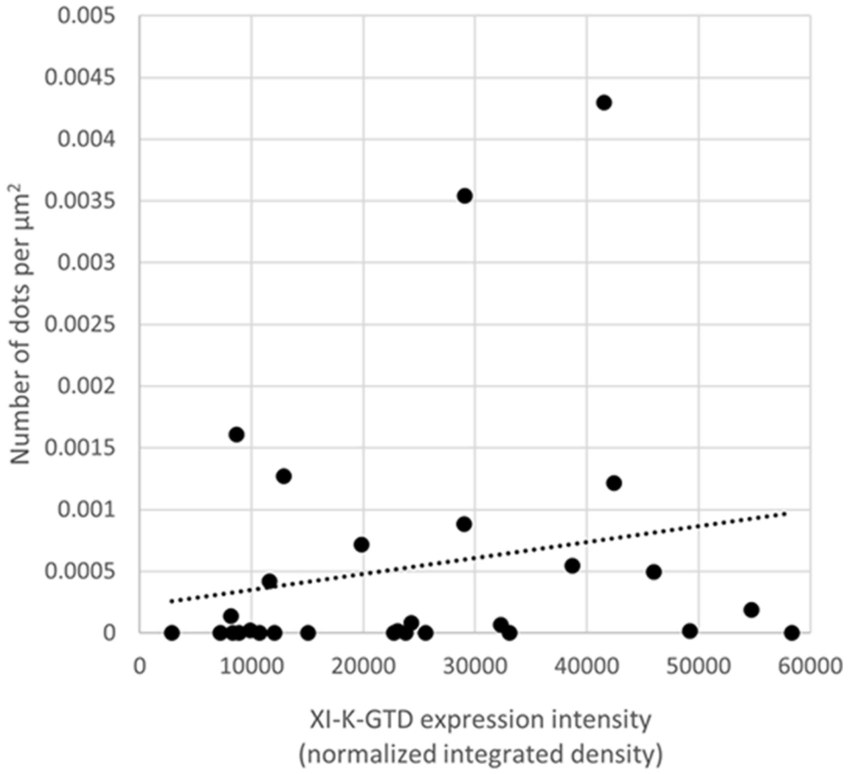

Figure S1. Expression and localization of XI-K-GTD.

(A) Expression intensity and number of dot-like structures of the GTD construct was measured in Col-0 midvein cells transiently expressing YFP-XI-K-GTD.
