# Supplementary material for: Unravelling the molecular basis of the dominant negative effect of myosin XI tails on P-bodies
Source: PLoS One. 2021 May 26;16(5):e0252327. doi: 10.1371/journal.pone.0252327 (PMC8153422; doi:10.1371/journal.pone.0252327)
Supplement: S2 Fig — Expression intensity of XI-K-GTD and DCP1 was measured in Col-0 midvein cells transiently expressing YFP-XI-K-GTD and DCP1-CFP with or without XI-KΔGTD-DCP2. The error bars depict the standard deviation. Significance was determined with a two-sided t-test at p<0.001 (***), p<0.01 (**), and p<0.05 (*). (PDF) [file pone.0252327.s002.pdf]

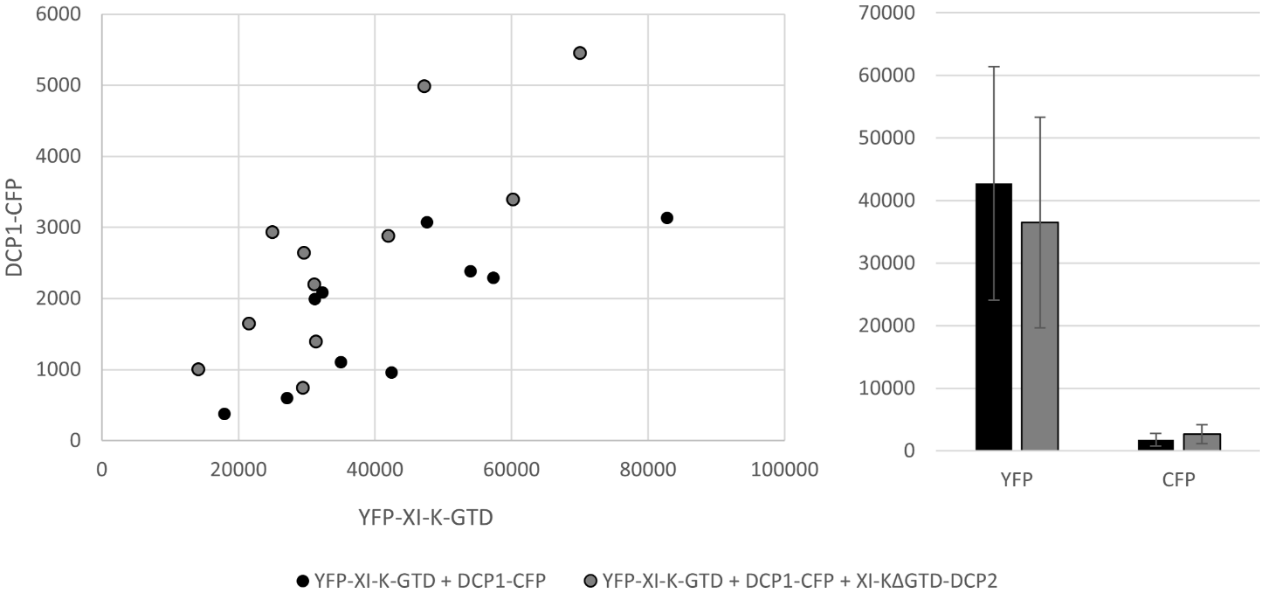

Figure S2. Expression and localization of XI-K-GTD.

Expression intensity of XI-K-GTD and DCP1 was measured in Col-0 midvein cells transiently expressing YFP-XI-K-GTD and DCP1-CFP with or without XI-$K_{\Delta GTD}$-DCP2. The error bars depict the standard deviation. Significance was determined with a two-sided t-test at p<0.001 (***), p<0.01 (**), and p<0.05 (*).
