# Supplementary material for: Unravelling the molecular basis of the dominant negative effect of myosin XI tails on P-bodies
Source: PLoS One. 2021 May 26;16(5):e0252327. doi: 10.1371/journal.pone.0252327 (PMC8153422; doi:10.1371/journal.pone.0252327)
Supplement: S1 Table — (PDF) [file pone.0252327.s003.pdf]

Table S1. Primer list

Gene (ID)	Primer forward	Primer reverse
XI-1 CC (AT1G17580)	GGGGACAAGTTTGTACAAAAAAGCAGGCTCAATGC TGAAAATGGCTGCAAGAGACACCGGT	GGGGACCACTTTGTACAAGAAAGCTGGGTA TCMCGGTGAGATGGTAAGAGTTTGTTGC
XI-1 GTD (AT1G17580)	GGGGACAAGTTTGTACAAAAAAGCAGGCTCAATGA AATCGCTTAATCAGAAACAGCAG	GGGGACCACTTTGTACAAGAAAGCTGGGTA TCMATCTGACCTTTCCAACAAGAACATGA
XI-2 CC (AT5G43900)	GGGGACAAGTTTGTACAAAAAAGCAGGCTCACTTAA GATGGCTGCTAGAGAAACTGG	GGGGACCACTTTGTACAAGAAAGCTGGGTA TCMAGGAGTGCTTATTGTTTGCTGATGCA
XI-2 GTD (AT5G43900)	GGGGACAAGTTTGTACAAAAAAGCAGGCTCAGGGA AATCTGCTGCAGAACGTCAAAT	GGGGACCACTTTGTACAAGAAAGCTGGGTA TTAGTGCAAGAATACAAATGCTGGA
XI-I CC (AT4G33200)	GGGGACAAGTTTGTACAAAAAAGCAGGCTCAATGC TTAAACAGGTTGCTAATGAAGCAG	GGGGACCACTTTGTACAAGAAAGCTGGGTA TCMTGGGGATGTAATTAGCGTCTTTTGCA
XI-I GTD (AT4G33200)	GGGGACAAGTTTGTACAAAAAAGCAGGCTCAATGA AATTAACCGCAGAAAGAAACCTGGA	GGGGACCACTTTGTACAAGAAAGCTGGGTA TCMAATGATCTGCTTTGAGGTTGAAGCTA
XI-K CC (AT5G20490)	GGGGACAAGTTTGTACAAAAAAGCAGGCTCAATGC TTAAGATGGCCGCACGAGACACAG	GGGGACCACTTTGTACAAGAAAGCTGGGTA TCMTGGCGAAATAGCAAGTGCCTGTTGACG
XI-K GTD (AT5G20490)	GGGGACAAGTTTGTACAAAAAAGCAGGCTCAATGG AAAAGCAACAGGAAAACCAGGA	GGGGACCACTTTGTACAAGAAAGCTGGGTA TCMTCCCGATGTACTGCCTTCTTTACGTGT
DCP1 (AT1G08370)	GGGACAAGTTTGTACAAAAAAGCAGGCTTAATGTCT CAAAACGGGAAGAT	GGGGACCACTTTGTACAAGAAAGCTGGGTTT WATTGTTGAAGTGCATTTT
DCP2 (AT5G13570)	GGGGACAAGTTTGTACAAAAAAGCAGGCTTAATGTC GGGCCTCCATCGATC	GGGGACCACTTTGTACAAGAAAGCTGGGTTT CMAGCTGAATTACCAGATT
DCP5 (AT1G26110)	GGGACAAGTTTGTACAAAAAAGCAGGCTTAATGGC GGCTGATAATACGGG	GGGGACCACTTTGTACAAGAAAGCTGGGTTT WAGGTAGTACGATTTGATA
VCS (AT3G13300)	GGGGACAAGTTTGTACAAAAAAGCAGGCTTAATGG CGTCTTCTCCTGGTAA	GGGGACCACTTTGTACAAGAAAGCTGGGTTT WATTTGCAACCCATAAGCA
XRN4 (AT1G54490)	GGGGACAAGTTTGTACAAAAAAGCAGGCTTAATGG GAGTACCGGCGTTCT	GGGGACCACTTTGTACAAGAAAGCTGGGTA TCACAAGTTTGCACCTCGATGACT
LifeAct-CFP	GCCGCACTCGAGATGGGTGTCGCAGATTTGATCAA GAAATTCGAAAGCATCTCAAAGGAAGAAGGGGATC CACCGGTCGCCACCATGGTGAGCAAGGGCGAGGA	GCTGGGTCTAGATTACTTGTACAGCTCGTCC A
