# Supplementary material for: Unravelling the molecular basis of the dominant negative effect of myosin XI tails on P-bodies
Source: PLoS One. 2021 May 26;16(5):e0252327. doi: 10.1371/journal.pone.0252327 (PMC8153422; doi:10.1371/journal.pone.0252327)
Supplement: S2 Table — Significance was determined with a two-sample test at p<0.001 (***), p<0.01 (**), and p<0.05 (*). (PDF) [file pone.0252327.s004.pdf]

Table S2. Wilcoxon test with multiple testing correction of P-body motility in myosin single mutants and under the dominant negative effect of myosin fragments in *Arabidopsis thaliana* leaf midvein cells.

Significance was determined with a two-sample test at $p<0.001$ (***), $p<0.01$ (**), and $p<0.05$ (*).

	Col-0	*xi-1*	*xi-2*	*xi-i*	*xi-k*	XI-1 CC	XI-2 CC	XI-I CC	XI-K CC	XI-1 GTD	XI-2 GTD	XI-I GTD	XI-K GTD
Col-0		**	ns	ns	***	**	**	***	**	***	***	***	***
xi-1	**		ns	ns	***	ns	ns	ns	ns	***	**	***	***
xi-2	ns	ns		ns	***	ns	ns	ns	ns	***	***	***	***
xi-i	ns	ns	ns		***	ns	ns	*	ns	***	***	***	***
xi-k	***	***	***	***		***	***	***	***	***	ns	***	ns
XI-1 CC	**	ns	ns	ns	***		ns	ns	ns	***	ns	***	***
XI-2 CC	**	ns	ns	ns	***	ns		ns	ns	***	ns	***	***
XI-I CC	***	ns	ns	*	***	ns	ns		ns	***	ns	***	***
XI-K CC	**	ns	ns	ns	***	ns	ns	ns		***	ns	***	***
XI-1 GTD	***	***	***	***	***	***	***	***	***		***	***	ns
XI-2 GTD	***	**	***	***	ns	ns	ns	ns	ns	***		***	***
XI-I GTD	***	***	***	***	***	***	***	***	***	***	***		***
XI-K GTD	***	***	***	***	ns	***	***	***	***	ns	***	***	
