# Supplementary material for: Unravelling the molecular basis of the dominant negative effect of myosin XI tails on P-bodies
Source: PLoS One. 2021 May 26;16(5):e0252327. doi: 10.1371/journal.pone.0252327 (PMC8153422; doi:10.1371/journal.pone.0252327)
Supplement: S3 Table — Significance was determined with a two-sample test at p<0.001 (***), p<0.01 (**), and p<0.05 (*). (PDF) [file pone.0252327.s005.pdf]

Table S3. Wilcoxon test with multiple testing correction of P-body speed data, collected by manual tracking of DCP1-CFP marked P-bodies in transiently transformed *Arabidopsis thaliana* leaf midvein cells.

Significance was determined with a two-sample test at p<0.001 (***), p<0.01 (**), and p<0.05 (*).

	Col-0	3KO	3KO + XI-K	3KO + XI-K$_{\Delta GTD}$-DCP2	3KO + XI-K$_{\Delta GTD}$-DCP5	3KO + XI-K$_{\Delta GTD}$-VCS	3KO + XI-K$_{\Delta GTD}$-XRN4
Col-0		***	**	ns	***	***	***
3KO	***		***	***	***	***	***
3KO + XI-K	**	***		ns	ns	ns	ns
3KO +XI-KΔGTD-DCP2	ns	***	ns		***	***	***
3KO +XI-KΔGTD-DCP5	***	***	ns	***		*	ns
3KO +XI-KΔGTD-VCS	***	***	ns	***	*		ns
3KO + XI-KΔGTD-XRN4	***	***	ns	***	ns	ns	
